# Understanding the Self in Individuals with Autism Spectrum Disorders (ASD): A Review of Literature

**DOI:** 10.3389/fpsyg.2017.01422

**Published:** 2017-08-22

**Authors:** Ann X. Huang, Tammy L. Hughes, Lawrence R. Sutton, Marissa Lawrence, Xiaohan Chen, Zhe Ji, Waganesh Zeleke

**Affiliations:** ^1^Department of Counseling, Psychology and Special Education, School of Education, Duquesne University, Pittsburgh PA, United States; ^2^Director of International Students Affairs, Vincennes University, Vincennes IN, United States

**Keywords:** the self, self-awareness, high-functioning autism, adolescence, Asperger syndrome, theory of mind deficit, social communication deficits

## Abstract

When the system of self is explored in individuals with Autism Spectrum Disorders (ASDs), it is important to measure it via both their own perceptions of the self and their understanding of others’ perceptions on themselves at a multidimensional level. This paper reviews existing research in this area using a three-dimension approach. Researchers have found that impairments in the self-system are usually correlated with these individuals’ social and cognitive functioning levels: high functioning individuals with ASD who have higher IQ are found to have better awareness of their limitations in social and communication domains than those with lower IQ. Many researchers believe that there *are* impairments in the psychological (but not physical) self in individuals with ASD, such as theory of mind deficits due to social and communicative impairments. On the other hand, some researchers argue that individuals with ASD have *selective* rather than *global* impairments in the self. In other words, the impairment usually lies in a specific aspect of functioning in individuals with ASD. Insights from the review of existing literature on this topic may be able to shed some lights on the development of effective intervention programs to improve social communication deficits in this population.

## Introduction

### Self-Image/Concept

The self system is a multi-dimensional construct where self-image/concept is an important component (Peterson et al., 1984). Self-image (also called self concept in some literature) is defined as one’s experiences and ideas about the self in various facets of life (Coombs, 1981; Peterson et al., 1984). The insights of important others and one’s experiences of these perspectives is likely to affect a person’s self-image/concept (Rosenberg, 1979). Offer et al. (1981) believed an individual’s functioning in various social (e.g., school, family, and peer groups) and psychological contexts (e.g., impulse control, mental health adjustment, ease in new situations) are the primary domains reflected in self-image. Thus, they proposed that a complete measurement of one’s self-image/concept should be multidimensional.

To assess the total concept of self, it is important to measure one’s understanding of both one’s own perceptions and those of others, as well as different social and psychological contexts from which others’ perceptions are coming (Williams, 2010; Zahavi, 2010). According to the socioecological theory (Germain and Bloom, 1999), both social and physical environments have an important role to play in human behaviors as behavior is learned. Based on this theory, we continuously form relationship with people and environments surrounding us via reciprocal exchange/interactions. During this process, our behaviors may be influenced, shaped, and even changed. We observe and learn from others’ behaviors and regulate own behaviors accordingly. Thus, human beings form self-image through interactions with both people and environments surrounding them. (Wehmeyer and Shogren, 2008).

### Self-Awareness in Individuals with ASD

Self-awareness is an important terminology under self-concept. Self-awareness refers to being aware of self as “the object of own attention,” including “one’s own mental states (e.g., perceptions, sensations, attitudes, intentions, emotions), public self-aspects (e.g., behaviors [including actions—Knoblich and Flach, 2003], and general physical appearance)” (Morin, 2004, p. 198). Morin (2006) suggests there are four levels of self-awareness: unconsciousness, consciousness, self-awareness and meta-self-awareness. Self-awareness has been studied among various clinical groups including individuals with Autism Spectrum Disorders (ASD). Individuals with ASD are mainly characterized by impairments in (1) language and communication, (2) reciprocal social interaction, and (3) repetitive behaviors, intense interests and sensory dysfunction (American Psychiatric Association, 1994). Due to the heterogeneous nature of the condition, self-awareness can be a unique experience for each person on the spectrum (Elmose, 2016).

Elmose (2016) described a conceptual framework for understanding characteristics of self-awareness in individuals with ASD. Considering the characteristics of ASD, examples of self-awareness experiences in individuals with ASD within this framework include the following: (1) they do not know what they do not know, so it is hard for them to judge when and how to know more; (2) they have difficulty telling the differences between their own or others’ preferences and emotions in social situations; (3) they have difficulty relating their own behaviors to environmental and social contexts/situations, and to others’ actions; (4) they have difficulty understanding self and others’ thoughts and feelings, etc. He summarized various theoretical positions in his review and concluded that there are differences in self-awareness at all level (except unconsciousness), but these differences mainly occur in the psychological (but not physical) domain of the self.

## Major Characteristics of ASD and Impacts on the Self

Kanner (1943) published the first research paper in autism. One year later, Hans Asperger reported a few higher functioning cases with similar symptoms. The original term *autism* came from the Greek *autos*, which means “self” (Elmose, 2016). In both Kanner and Asperger’s descriptions of their original cases with ASD, they both addressed differences in understanding the self in their original cases (Elmose, 2016). Subsequent research from various sources (including clinical descriptions, autobiographies, self-reports, and parent reports) also suggested that differences in self-awareness might be linked to the difficulties individuals with ASD experience daily. The following section describes the major difficulties individuals with ASD usually face and how these difficulties impact the self in this population.

### Difficulty with Transition/Changes

Individuals with ASD typically do not respond well to transition/changes (American Psychiatric Association, 1994). As individuals with ASD grow, they, like other typically-developing (TD) adolescents, face significant changes in school settings. Changes in location, schools, social contexts, peer groups, and family interactions may have negative effects on the daily functioning of individuals with ASD, or on their attitude toward certain situations. Sometimes these changes may even intensify at-risk issues (Schreiber, 2011). For example, adolescence brings larger and more complex physical environments (e.g., building size and classroom options when compared to elementary school) and more socially complex and demanding peer relations. Due to their social and communication deficits, addressing social problems (e.g., difficulty in making and keeping friends) are more evident in this group during adolescence than childhood. Previous research has found that emotional distress (e.g., feelings of loneliness, anxiety, and depression) is associated with poor social adjustment and/or social isolation in adolescents with ASD (Attwood, 1998; Gillott et al., 2001; Bauminger et al., 2003). Unavoidably, these emotional issues have negative impacts on their self-image (Peterson et al., 1984).

### Joint Attention, Language, and Theory of Mind Deficits

Joint attention, language, and social communication skills are common challenges faced by individuals with ASD, which can lead to difficulties in social relationships and relating to peers. Generally, individuals with ASD are not proficient in using language as a tool for guiding behavior and emotional regulation (Rubin and Lennon, 2004), which may lead to uncomfortable or awkward social situations when interacting with others, and consequentially cause ineffective social communication in this population. Additionally, individuals with ASD have difficulty perceiving emotional states and considering plausible causal factors, initiating and maintaining conversational exchanges, understanding the interests of others and previous knowledge others hold (Rubin and Lennon, 2004), which may cause misconceptions about others. Furthermore, poor social problem solving skills suggests they cannot recognize and repair breakdowns in these exchanges (Rubin and Lennon, 2004), or appropriately perceive emotional reactions of others (i.e., theory of mind deficits–failure to understand the perspective of others in daily situational interactions; Baron-Cohen, 1995; Schreiber, 2011).

It is noted that individuals with ASD also struggle with expressing and communicating their own thoughts and emotions (Dritschel et al., 2010). Friends, family, and school personnel may not clearly comprehend their messages, which may result in an unfulfilling response, and eventually followed by problematic social isolation. Offer et al. (1981) believed a person’s functioning in various social and psychological domains reflects one’s self-image, thus problematic social isolation somehow mirrors negative self-image in this population. Lack of age-appropriate play and deficits in social communication exchange (Baron-Cohen, 1997) may lead peers to perceive these individuals as unintelligent, aloof, or odd (Craig and Baron-Cohen, 1999).

In addition, researchers have found that verbal mediation (i.e., inner speech) in individuals with ASD is somehow atypical when compared to typically developing peers (Lidstone et al., 2009; Williams and Jarrold, 2010; Williams et al., 2012). According to Vygotsky’s 1934/1987 influential theory, one’s ability to “think in speech” (i.e., inner speech, or internalized self-talk) is a critical way to regulate one’s own behaviors, without the presence of others (Morin, 2005; Williams et al., 2012). Researchers believed the atypical development of inner speech in individuals with ASD may prevent them from regulating their own behavior under various social contexts (Williams et al., 2012). In summary, deficits in the domains mentioned above may have negative impacts on self-perception in this population.

### Abstract Reasoning

Solomon et al. (2011) found that individuals with ASD also demonstrate difficulty with abstract reasoning. Abstract reasoning is defined as “an element of executive functioning that requires the considerations and manipulation of information about events, objects, and concepts that are not in the immediate environment” (Solomon et al., 2011, p. 32). Abstract reasoning includes concept identification and formation, as well as organization of information. Individuals who lack abstract reasoning skills also tend to have difficulty understanding the intent of the actions they observe. Because social interactions are often formed by mirroring and imitation of others, if one fails to demonstrate or engage in appropriate and reciprocal social behavior, quality interpersonal relationship is not likely to develop (Solomon et al., 2011). Deficits in the pragmatic aspects of language are also found associated with difficulty in abstract reasoning in this population (Solomon et al., 2011). In summary, abstract reasoning deficits have negative impacts on daily social functioning in individuals with ASD. Since an individual’s functioning in various social and psychological domains reflects one’s self-awareness (Offer et al., 1981), abstract reasoning deficits may have a negative impact on self-awareness in individuals with ASD.

### Understanding of Friendship

Friendship is critical for human development. It is an important variable in moral, social, and emotional growth. Companionship, intimacy-trust, and affection are the three basic functions of friendship, which also separate friends from non-friends for older children and adolescence (Parker and Gottman, 1989; Buhrmester, 1990). The quality of friendship is directly related to the sense of the self: healthy friendships also serve as a protective factor from developing psychopathology (Solomon et al., 2011).

Individuals with ASD tend to have difficulties conceptualizing or verbalizing their understanding of what constitutes a friend or issues related to friendship (Carrington et al., 2003). It is important to note that youths with ASD are apt to engage in friendships that are different in the duration, frequency of meetings, and types of activities they enjoyed together when compared to typically developing peers (Bauminger et al., 2008). Solomon et al. (2011) found that various levels of social functioning deficits in individuals with ASD limit such relationships, consequentially limiting healthy social interaction with others. This may contribute to emotional issues such as social anxiety, depression, loneliness (Attwood, 1998; Gillott et al., 2001) and social isolation (Bauminger et al., 2004). These social and emotional problems can have negative impacts on the mental health wellbeing and sense of self in individuals with ASD.

### Deficits in Reciprocal Social Communication

Due to the lack of meaningful social experiences and theory of mind deficits mentioned earlier, individuals with ASD tend to have great difficulty understanding complex reciprocal social communication. This may lead to misunderstanding of other people’s attitudes toward themselves in individuals with ASD. For example, usually, when one is aware of that other people have a negative attitude toward oneself, this will have a negative impact on one’s self-concept; however, if one is not aware of that, or even misunderstands that as a positive attitude, this may limit one’s motivation to make any significant self-improvement (Dritschel et al., 2010).

In summary, self-awareness is an individual experience so it is unique to everyone. Understanding of the self can greatly vary in individuals with ASD due to the heterogeneous nature of the condition. It can be affected by their levels of cognitive functioning and adaptive behaviors.

## Review of Literature on the Self System in Individuals with ASD

Numerous studies have focused on the self-system of typically developing children or adolescents can be found in current literature. However, research on the same topic involving individuals with disabilities has been limited. In particular, research on the self system involving children or adolescents with ASD has been scarce.

### Criteria for Inclusion

Studies selected for this review include journal articles and books (or book chapters) that: (a) were either qualitative case studies or larger scale exploring quantitative studies that involve individuals with ASD and the self-system; (b) were reviews of literature that involve individuals with ASD and the self-system; (c) were published between 1980 to 2017 in peer-reviewed journals or books in the fields of ASD (e.g., *Autism, Focus on Autism and Other Developmental Disabilities*, and *the Journal of Autism and Developmental Disorders*, etc.), psychology (e.g., *Child Development* and *North American Journal of Psychology* and *Journal of Child Psychology and Psychiatry*), and psychiatry (*e.g.*, *Development and Psychopathology* and *the British Journal of Psychiatry)*, or other disability-related areas (e.g., *Journal of Developmental and Physical Disabilities* and *Journal of Developmental Disabilities*).

### Procedure

Articles and books were located by searching a series of online database (PsycINFO, ERIC, InfoTrac, ProQuest, PubMed, and google scholar, etc) by using ASD or Asperger syndromes (AS) and one of the following key words respectively: self, self-image, self-understanding, self-esteem, self-awareness, or self-efficacy, etc. Once an article was located, the authors would take an initial read to determine whether or not it was relevant and appropriate for this topic before being selected to be used in the review.

### Three Dimensions of the Self-system

The self system is a complex concept that involves “a multitude of notions of the self” (Zahavi, 2010, p. 548). When investigating the “multifaceted character of self,” one has to be able to tell the difference between “types of self-awareness,” and understand “how they relate to each other” (Zahavi, 2010, p. 549). In general, current research in the field mainly explores the self in individuals with ASD from one of the following three dimensions: an *experiential* dimension, an *interpersonal* dimension and a *narrative* dimension (Zahavi, 2010).

Early research aimed at identifying a general impairment of self-experience and self understanding in individuals with ASD falls under the category of *experiential* dimension. Under this framework, the mechanism that one accesses to one’s own mind is the same as one employs to understand mental states of others, which means, the same mediating theory goes for both self and other (Carruthers and Smith, 1996; Frith and Happé, 1999). For example, participants with a diagnosis of ASD were asked to complete a standardized protocol to examine their performance on a certain task, then comparison will be made with typically developing peers on the same task to see how the two groups perform differently. However, this approach has been criticized recently due to conflicting research findings (Zahavi, 2010). For example, in Baron-Cohen’s (1989) earlier publication, he believed children with autism were mind-blind and had little awareness of their own mental states; but then in his later work, he stated that children with classic autism singularly focus on the self (Baron-Cohen, 2005).

More recent research, on the other hand, takes on the *interpersonal* dimension method targeting specific deficits related to the self (Zahavi, 2010). Technically, it refers to a dimension of self that is basically reflected by, or mediated through, the perspectives of others. In other words, it focuses on “the capacity to understand and adopt the evaluative perspectives of others on oneself,” and “to that extent, impairments on the level of interpersonal engagement will have ramifications for the subsequent development of various aspects of self” (Zahavi, 2010, p. 550). For example, as mentioned previously, when an individual with ASD shows impairments in recognizing others’ facial expressions (i.e., emotional states), he/she will not know how to respond appropriately to match the social situation, which may result in peer rejection (Bauminger et al., 2003). Repeated social failures are associated with emotional issues such as social anxiety or depression (Bauminger et al., 2003). This will definitely have a negative impact on the development of self in individuals with ASD.

The third approach is focused on the *narrative* dimension of self. Basically, according to the narrative approach, information about the self is collected via self narrative, from which we can synthesize “the diverse and heterogeneous aspects of life” and coordinate “different temporal dimensions,” as well as establish “a web of semantic relations that link past, present and future events into a meaningful whole”; the narrative self includes investment of others, just like “the story of any individual life is always interwoven with the stories of others” (Zahavi, 2010, p. 551). When this approach is used to explore the self in individuals with ASD, their own voice can be better heard.

In summary, the three dimensions are approaches that can be used to analyze various aspects of the self-system in individuals with ASD from a philosophical perspective. They are interrelated, and individuals with ASD can have selective impairment. The following section provides a brief review of existing literature exploring various aspects of the self involving individuals with ASD using the above three approaches.

#### Experiential Dimension Approach

Capps et al. (1995) conducted the first comparative study examining the relationships between perceived self-competence (i.e., self-perception of personal worth and efficacy), cognitive ability, understanding of emotional states, and parent report of social adaptation in 18 preadolescents and adolescents with high-functioning autism (HFA) and 20 comparison peers. The participants were asked to complete the *Perceived Competence Scale for Children* (SPPC, Harter, 1985) and to identify the four emotional states presented to them. In addition, parents of children with HFA were asked to complete *the Vineland Adaptive Behavior Scales* (Sparrow and Cicchetti, 1989) to find out their children’s social and adaptive competencies in the following four domains: communication, daily living skills, socialization, and motor skills.

Results suggested that overall participants with HFA perceived themselves to be less competent than their comparison peers in all but the cognitive domain. When correlating with IQ, results indicated that, in the HFA group, participants with higher IQ scores seemed to perceive themselves as less competent in the social domain than those with lower IQ scores, but have better understanding of emotion in self and others. However, in the comparison group, IQ was found to be positively correlated with their perceived cognitive but not social competence. Parent reports showed that in the HFA group, participants with higher IQ who perceived themselves less social competence displayed less emotional distress, such as sadness or fear, than those who have lower IQ but perceived greater social competence. Researchers concluded that HFA participants with higher IQ who were able to better understand emotions might have greater self-awareness allowing them to see the difference (e.g., social limitations) between themselves and typically developing peers.

A later study that sought to replicate the above study found slightly different results. Vickerstaff et al. (2007) also used the SPPC to investigate self-perceptions of social limitations and relationship between self-perceived social deficits and depression in 22 children and adolescents with HFA. Results also confirmed Capps et al. (1995)’s findings that the higher IQ the participants have, the lower level of self-perceived social competence, and vice versus. However, an important difference emerged showing that participants with higher IQ who displayed lower self-perceived social competence actually self reported higher level of depression symptoms, in contrast to the original study the parents reported fewer depression symptoms in HFA participants with higher IQ (Capps et al., 1995). The researchers reasoned that an increase in awareness of their own social communication deficits (e.g., unable to fit in their peers’ social world) and their inability to remedy these social situations may contribute to the development of such emotional distress including symptoms of depression and anxiety. Vickerstaff et al. (2007) also stressed that it is the child’s self-perceived social competence that relates to depression symptoms, not the actual level of their social competences as rated by their parents and teachers.

To examine the understanding of own inner mental states in adolescents with AS, Dritschel et al. (2010) compared 22 participants with AS and same number of typically developing peers. Results showed that adolescents with AS had more difficulties in this aspect of social functioning than their typically developing peers or than adults with the same condition (i.e., AS). Specifically, due to their awareness of own social skills limitation and their difficulty in understanding self inner mental states, adolescents with AS tended to “overestimate other people’s social competence” (p. 517). For example, adolescents with AS would consult “a comparison person to know more about their own inner mental states than themselves” (p. 515), suggesting “a poorly differentiated self-identity may underline social difficulties” (p. 517), and can cause emotional issues such as depression and anxiety. This is consistent with existing research findings (Attwood, 1998; Gillott et al., 2001; Bauminger et al., 2003).

A recent Japanese study (Yoshimura and Toichi, 2014) yielded similar findings. They examined self-consciousness (a type of self-awareness) in 18 adolescents with AS, 19 with PDD-NOS and 19 typically developing as control group using an episodic memory task (i.e., self-reference effect). Results showed that both the PDDNOS group and the control group did very well, but the AS group did not. The researchers found that both ASD groups (i.e., PDDNOS and AS) showed an atypical pattern of relationship between memory performance and IQ. Based on the results, the researchers believed that unlike individuals with AS, individuals with PDDNOS may have the same level of self-consciousness as their typically developing peers. However, maybe due to their unique cognitive process (e.g., being aware of what was going on around them but unable to self-regulate own behaviors), the way of being self-conscious in this group seemed atypical, which lead to social impairments similar to those experienced by the AS group. The researchers also pointed out the relationship between psychiatric disorders and the level of self-consciousness in ASD. As generally recognized (e.g., Mazurek and Kanne, 2010), the higher IQ a person with ASD has, the more insight and self-awareness of own deficits in three core areas (i.e., social, communication and behavior domains) he/she has, which can cause anxiety/depression. Thus, it is not surprising that individuals with high functioning ASD show more anxiety/depression symptoms than those who are low functioning on the spectrum.

Similarly, Elmose and Happé (2014) examined accuracy of judging own memory performance in response to social vs. non-social stimuli in a group of children with ASD in comparison to a group of typically developing (or TD) children. Results indicated comparable levels and patterns of accuracy in the ASD and TD groups, with ASD group being more accurate in judging own memory for non-social than social stimuli, and the opposite pattern for the TD group.

Schriber et al. (2014) examined personality and self-insight in individuals with ASD. They first compared Self-Report of Big Five personality traits in adults with ASD and TD controls, then in children/adolescents. Results suggested that personality differences between ASD and TD individuals (1) were evident in both children/adolescents and adulthood; (2) were similar for men and women; (3) were found via self- and parent report. Compared to their TD peers, individuals with ASD tended to more Neurotic and less Extraverted, Agreeable, Conscientious, and Open to Experience. Researchers also found that these personality trails predict ASD diagnosis well. The researchers also assessed the level of self-insight in these two group by examining the differences in self-other agreement and self-enhancement (vs. self-diminishment) biases (in the personality protocol mentioned above) by comparing the data collected via self-report and parent reports. The study failed to find significant difference in self-insight. However, results suggested that although individuals with ASD had similar level of insight into self-other agreement to TD control group, individuals with ASD had a tendency to self-enhance, while TD individuals to self-diminish.

#### Interpersonal Dimension Approach

In a study examining the relationship between social acceptance, internalizing and externalizing problem behaviors, perceived social skills and friendships, Viecili et al. (2010) collected data from 40 children and adolescents with autism via self report, where parents were interviewed for the number of friends their child had. Results showed that social acceptance was positively correlated with social skills and number of friends in school and negatively correlated with internalizing behaviors including symptoms of anxiety, depression, or low self-esteem. Consistent with previous research, these findings indicate that greater social acceptance may also have a positive impact on self-concept of youth with developmental disabilities including autism (Weiss et al., 2003).

Another study aimed at measuring perceptions of social problems and adaptive behaviors in children and youth with AS yielded somewhat different but insightful findings. Barnhill et al. (2000) used the Behavior Assessment System for Children (BASC, Reynolds and Kamphaus, 1992) to investigate the perception difference between parents (parent rating scale), teachers (teacher rating scale), and student self (self-report of personality). A total of 20 individuals with AS participated in the study. Results revealed that there was significant difference in perceptions between parents and the teachers in most sub-domains. In general, parents reported high-level mental, emotional, and behavioral problems in their children. Teachers reported similar but much less severe symptoms. Results also showed the self perceptions of individuals with AS were distinctly different from parents’ and teachers’. Students themselves were not aware of having these problems (e.g., they perceived themselves to be similar to peers, or their responses were in the average range) and still felt positive about their general social skills. Researchers concluded that children/youth with AS lack self awareness or denial of their disability. Mazefsky et al. (2011) argued that although self-report measures by individuals with ASD may be able to offer valuable information to some extent, professionals who work with this population should not rely too heavily on self-report instruments for decision-making, better coupled with other data collection approaches such as observation or parent and/or teacher report, to get a more objective view.

#### Self-Narrative Approach

This section reviewed several studies using self-narrative approach to measure the self in individuals with ASD. Lee and Hobson (1998) used Damon and Harter’s *Self-Understanding Interview* (or SUI, 1988) to measure the self-concept of social experiences in children and adolescents with autism and intellectual disability (ID). Results from the SUI showed that compared to their peers with ID, participants with autism tended to talk less about their social experiences; however, they were able to describe themselves perfectly in physical, active, and even psychological terms. Researchers hypothesized that these individuals seemed to be less concerned about the issues of their social relationships and interactions with others. This indicated some individuals with ASD might have selective impairments in the psychological or interpersonal self, which were also referred to the so-called theory of mind deficits that prevented them from understanding their own social deficits (Baron-Cohen, 1995). Williams (2010) believed individuals with ASD would apply an alternative, compensatory mechanism to assist themselves in understanding mental states of own or of others when they have a selective theory of mind deficit, for example, via memorizing social or behavioral rules.

Farley et al. (2010) replicated the above study. They employed a modified version of Damon and Harter’s (1988) self-as-subject interview, and focused on narratives of 16 adolescents with HFA or AS and a comparison group of typically developing peers, aiming at examining their ability to conceptualize self through the perceptions of other. The participants were asked to conceptualize themselves from others’ perspectives. Results showed although there was no significant difference in the ability to self-conceptualize under both the categories of *continuity* and *distinctiveness* between adolescents with ASD and their comparison peers, the ASD group had more difficulty in self-conceptualization of *agency*, which was defined as “the information, influences, and control of the self” (Farley et al., 2010, p. 526). The researchers argued that only certain aspects of the self in individuals with ASD are impaired, and should be examined separately to fully understand the specific strengths and needs of individuals with ASD.

In a more recent study that examines self presentation skills of high-functioning children and adolescents with ASD (Scheeren et al., 2010), the participants were asked to describe themselves twice, first without any direction from audience (in this case, the audience were the researchers) as a baseline condition, then with audience preference and demands as a direction-guided condition. Results showed both participants with ASD and the comparison group tended to provide a more positive description about themselves under directed condition. However, the ASD group seemed to be less skillful in responding to the audience directions, compared to the comparison group. The researchers reasoned that this might be a result of their theory of mind deficits or their tendency to rigidly stick to moral and social rules.

## Conclusion

In conclusion, the review of the above literature has clearly shown that despite different research findings, there is consensus among researchers that there *are* impairments in the psychological self in individuals with ASD. Some researchers believed due to social and communicative impairments as well as theory of mind deficits, individuals with ASD generally have limited awareness in the self and others. Other researchers argue that they have *selective* rather than *global* impairments in the self (Zahavi, 2010). In other words, they believe the impairment usually lies in a specific aspect of functioning in individuals with HFA or AS. For example, when compared to typically developing peers, adolescents with HFA or AS are relatively weak in their ability to accurately engage in self-perception in social-emotional domains (Farley et al., 2010). They also have difficulty understanding the mental states of self and others, and tend to feel positive about themselves (e.g., Barnhill et al., 2000; Scheeren et al., 2010). Another example is when examining their self-conceptualization ability, individuals with ASD have difficulty conceptualizing interpersonal relations, and are unable to grasp certain social and interpersonal concepts such as friendship (Bauminger et al., 2004). Research has also found that many high-functioning individuals with ASD who have higher IQ may be aware of their social deficits, but unable to address them appropriately (Capps et al., 1995; Vickerstaff et al., 2007; Supplementary Table 1).

Insights into the characteristics and mechanism of the self in individuals with ASD will allow service providers to develop interventions to help this population better handle challenges in life (Elmose, 2016). For example, insights from research in this topic may be able to shed some lights on the development of effective intervention programs to improve social communication deficits in this population. Systematic social skill training targeting specific deficits may be offered to individuals with ASD to improve self-competence and eventually quality of life. In addition, individuals with ASD should have more opportunities to practice social skills (e.g., via modeling and role play) in various social contexts and be offered feedback on their behaviors, since human beings usually acquire self-awareness and knowledge by interacting with their environments (Wehmeyer and Shogren, 2008; Wehmeyer et al., 2010; Duff and Flattery, 2014).

## Author Contributions

AH is mainly responsible for the research idea, organization and writing the majority of the manuscript. TH and LS wrote the first part of the manuscript regarding the self system; ML and XC did the literature research and organized the literature in initial writing stage; ZJ and WZ did further analysis of the literature and revised the work that was done by ML and XC.

## Conflict of Interest Statement

The authors declare that the research was conducted in the absence of any commercial or financial relationships that could be construed as a potential conflict of interest.
