# Supplementary material for: Understanding the Self in Individuals with Autism Spectrum Disorders (ASD): A Review of Literature
Source: Front Psychol. 2017 Aug 22;8:1422. doi: 10.3389/fpsyg.2017.01422 (PMC5572253; doi:10.3389/fpsyg.2017.01422)
Supplement: Supplementary file 1 [file Table_1.DOCX]

| Approach | Authors/Year | Research Focus | Results/Findings |
| --- | --- | --- | --- |
| Experiential | Capps et al. (1995) | Examined the relationships between perceived self-competence, cognitive ability, understanding of emotional states, and parent report of social adaptation in individuals with high functioning ASD. | 1. Participants with higher IQ self-perceived less competent in social domain than those with lower IQ; 2. Participants with higher IQ had greater self-awareness but less depression symptoms. |
|  | Vickerstaff et al. (2007) | Investigated self-perceptions of social limitations and relationship between self-perceived social deficits and depression. | 1. Participants with higher IQ had lower level of self-perceived social competence, and vice versus; 2. Participants with higher IQ had higher level of depression symptoms. |
|  | Dristschel et al. (2010) | Examined understanding of own inner mental states in adolescents with AS. | Adolescents with AS had more difficulties understanding own inner mental states than TD peers or adults with AS. |
|  | Yoshimura & Toichi (2014) | Examined self-consciousness in adolescents with AS and PDD-NOS using an episodic memory task. | 1. Both ASD groups showed an atypical pattern of relationship between memory performance and IQ: higher IQ, higher level of self-consciousness;  2. Confirmed the relationship between psychiatric disorders and the level of self-consciousness in ASD. |
|  | Elmose & Happé (2014) | Examined accuracy of judging own memory performance in response to social vs. non-social stimuli in children with ASD. | 1. Comparable levels and patterns of accuracy in the ASD and TD groups were found; 2. ASD group were more accurate in judging own memory for non-social than social stimuli, and the opposite pattern for the TD group. |
|  | Schriber et al. (2014) | Examined personality and self-insight in individuals with ASD using . | 1. Personality differences between ASD and TD individuals (1) were evident in both children/adolescents and adulthood; (2) were similar for men and women; (3) were found via self- and parent report; 2. Although individuals with ASD had similar level of insight into self-other agreement to TD control group, individuals with ASD tende to self-enhance, while TD individuals to self-diminish. |
| Interpersonal | Viecili et al. (2010) | Examined the relationship between social acceptance, internalizing and externalizing problem behaviors, perceived social skills and friendships. | 1. Social acceptance was positively correlated with social skills and number of friends in school and negatively correlated with internalizing behaviors including symptoms of anxiety, depression, or low self-esteem;  2. Greater social acceptance may also have a positive impact on self-concept in individuals with ASD. |
|  | Barnhill et al. (2000) | Measured perceptions of social problems and adaptive behaviors in children and youth with AS using BASC via parent and teacher rating scales as well as student self-report. | 1. Parents reported high-level mental, emotional, and behavioral problems in their children than teacher; 2. Students themselves were not aware of having these problems and still felt positive about their general social skills. |
| Self-Narrative | Lee & Hobson (1998) | Measured the self-concept of social experiences in children and adolescents with autism and intellectual disability (ID) using *Self-Understanding Interview.* | 1. Compared to their peers with ID, participants with ASD tended to talk less about their social experiences but talked perfectly in other domains; 2. They seemed to be less concerned about the issues of their social relationships and interactions with others, indicating some of them might have selective impairments in the psychological or interpersonal self. |
|  | Farley et al. (2010) | Examined individuals with ASD’s ability to conceptualize self through the perceptions of other using the revised *Self-Understanding Interview.* | 1. The ASD group had more difficulty in self-conceptualizing “the information, influences, and control of the self” (p.526); 2. Only certain aspects of the self in individuals with ASD are impaired. |
|  | Scheeren et al. (2010) | Examined self-presentation skills of high-functioning children and adolescents with ASDs. | 1. Both participants with ASD and the comparison group tended to provide a more positive description about themselves under directed condition; 2. ASD group seemed to be less skillful in responding to the audience directions might due to theory of mind deficits or their tendency to rigidly stick to moral and social rules. |
